# Prognostic value of modified-Gustave-Roussy Immunity Score in resectable proximal gastric cancer

**DOI:** 10.1097/MD.0000000000033334

**Published:** 2023-03-24

**Authors:** Yujing Shi, Mengyang Ju, Xiaoke Di, Xinchen Sun, Xiaojiao Chen, Chenhong He, Liang Liang

**Affiliations:** a Department of Oncology, Jurong People’s Hospital, Zhenjiang, China; b Department of Oncology, Jurong Hospital Affiliated to Jiangsu University, Zhenjiang, China; c Department of Radiation Oncology, Osaka University, Suita, Japan; d Department of Radiotherapy, First Affiliated Hospital of Nanjing Medical University, Nanjing, China.

**Keywords:** albumin, lactate dehydrogenase, modified-Gustave-Roussy Immunity (GRIm) Score, neutrophil lymphocyte ratio, prognosis

## Abstract

The prognostic evaluation of GRIm score has been confirmed in many tumor species. The purpose of this study is to clarify the value of GRIm score in the prognostic evaluation of patients with resectable proximal gastric cancer. A single center retrospective study was conducted in 174 patients with proximal gastric cancer who underwent radical total gastrectomy. An in-depth analysis was carried out to explore the prognostic differences between high and low GRIm, and the influencing factors of disease-free survival rates and overall survival rates were analyzed by Cox regression model and Kaplan–Meier method. A total of 174 patients were divided into two groups: 135 patients were marked in L-mGRIm and 39 patients in H-mGRIm groups respectively. The median OS of the H-mGRIm and L-mGRIm groups were 23.2 and 38.6 months, respectively. The median DFS of the H-mGRIm and L-mGRIm groups was 16.9 and 31.7 months, respectively. Both DFS and OS were significantly different between groups (*P* = .000, *P* = .000). In multivariate analysis, ZPS (2 vs 0–1: HR 1.99 95% CI 1.05–3.76 *P* = .035), LDH (≥193 vs <193:HR 0.6; 95% CI 0.38–0.95 *P* = .028), mGRIm score (2–3 vs 0–1: HR 2.4; 95% CI 1.09–5.23 *P* = .029) was independent risk factors of OS. The age (>65 vs ≤65 years HR 0.63; 95% CI 0.4–0.95 *P* = .003), LDH (>193 U/L vs ≤193 U/L: HR 0.55; 95% CI 0.37–0.82 *P* = .004) and mGRIm score (2–3 vs 0–1: HR 4.74; 95% CI 2.24–9.9 *P* = .000) as an independent risk factor for DFS. mGRIm score is a novel, simple and effective index for prognosis evaluation of resectable cardiac cancer and can be used as a part of the risk stratification process.

## 1. Introduction

Gastric cancer (GC) is the fifth most common malignancy in the world^[1,2]^and has become the third major cause of cancer-related deaths in recent years.^[3]^ The incidence rate of GC increases with age, and more than 1 million cases appear every year.^[4]^ Since most patients are in advanced stage at the time of diagnosis, the mortality of GC has continued to rise in the past decade, especially in China.^[5]^ About 20% of all GC cases are proximal GC, a malignant tumor occurring at the junction of stomach and esophagus. The mortality and recurrence rates of GC are high, which severely impact the life safety and quality-of-life of patients.^[6]^ Despite the progress in surgical technology and adjuvant therapy, the survival rate of GC patients is still very low.^[7]^ The tumor, node, metastasis (TNM) staging system is considered to be the best survival prediction standard, but in clinical practice, the clinical outcomes of patients with the same TNM stage and similar treatment are very different.^[8]^ This phenomenon also suggests that TNM staging alone cannot provide complete clinical information. Hence, more useful and effective prognostic evaluation indicators are needed to more accurately evaluate the prognosis of the patients, carry out reasonable treatment for high-risk patients and reduce the risks of recurrence and death.

Inflammation and nutrition play important roles in the survival and prognosis of cancer patients. The clinical indicators of inflammation include the neutrophil lymphocyte ratio (NLR), the lymphocyte to monocyte count ratio and C-reactive protein. These indicators are used to evaluate the survival and prognosis of tumor patients and are related to the prognosis of various cancers.^[9–12]^ Nutritional status^[13]^ and lactate dehydrogenase (LDH)^[14]^ are also reportedly related to tumor progression, invasion and metastasis, and can be used to evaluate the prognosis of tumor patients. Bigot et al constructed the GRIm score based on LDH, NLR, and albumin (ALB) as the inclusion standard for phase I clinical trials of patients with advanced tumors.^[15]^ The GRIm score is widely used in clinical immunotherapy or non-immunotherapy for various solid tumors, such as small cell lung cancer.^[16]^ non-small cell lung cancer^[12,17]^and esophageal cancer,^[18]^ and its role of prognosis evaluation has been confirmed. Studies show that a high GRIm score before treatment predicts worse overall survival (OS) and progression-free survival (PFS). The role of the GRIm score in evaluating the prognosis of patients suffered in proximal GC has never been studied. Hence, the purposes of this study were to retrospectively analyze the impact of preoperative GRIm score on the prognosis of proximal GC patients and to clarify the potential value of the GRIm score in proximal GC.

## 2. Data and Methods

### 2.1. Patients

Retrospective collection of proximal GC patients with total gastrectomy from June 2014 to July 2019, the final included 174 patients.

All data were taken from the electronic medical record system of our hospital. The inclusion criteria were: histological confirmation of cardiac adenocarcinoma; Zubrod – ECOG – performance status (ZPS) score of 0 to 2; blood routine and biochemical examinations 1 week before operation; reception of D1/D2 resection and negative margin (R0); complete medical records; age 18 to 80 years old; no distant organ metastasis. Exclusion criteria were: other malignant tumors; contraindications and no surgical treatment; incomplete clinical and follow-up data; preoperative chemotherapy or radiotherapy.

No patients with obvious infections, other chronic infectious diseases, and/or severe autoimmune diseases were included in this study.

### 2.2. Treatment and follow-up

All enrolled patients underwent surgery after complete preoperative evaluation. According to the seventh edition of AJCC guidelines,^[19]^ the pathologists classified TNM staging according to the depth of tumor infiltration and positive lymph nodes. The surgical methods included transabdominal or laparoscopic total gastrectomy combined with lymph node dissection, and adjuvant chemotherapy, which were determined according to the postoperative TNM staging and ZPS score. The commonly used chemotherapy regimens were Sox regimen (oxaliplatin + teggio) and DS regimen (docetaxel + cisplatin). There were totally 4 to 6 treatment cycles, each lasting 21 days, 6 patients did not receive postoperative adjuvant chemotherapy due to personal reasons. The clinical pathological parameters included age, gender, ZPS score, degree of differentiation, postoperative pathological stage, operation mode, nerve invasion, vascular tumor thrombus, and chemotherapy regimen. Laboratory parameters included neutrophil, lymphocyte and platelet counts, hemoglobin, ALB, and LDH. All patients signed the informed consents for treatment and for the use of clinical parameters. This study was approved by the ethics committee of our hospital.

The included patients were followed up regularly. The routine items concerned during this period included physical, laboratory and chest examinations combined with total abdominal computed tomography. The follow-up was up to December 01, 2021. The main results of interest included OS and disease-free survival (DFS). OS was defined as the survival time from the date of operation to the date of death from any cause, and DFS covered the survival time from the date of operation to the date of any cancer recurrence or distant metastasis.

### 2.3. Definition of GRIm score

GRIm score was proposed for the first time according to Bigot.^[15]^ The scoring standard is LDH ≤ normal range (225 U/L): 0 point, LDH > upper limit of normal value: 1 point; ALB ≥ 35 g/L: 0 point, ALB < 35 g/L: 1 point; NLR ≤ 6: 0 point, NLR > 6: 1 point. The total score ≤ 1 is defined as a low-GRIm score, and > 1 is defined as high -GRIm score.

### 2.4. Statistical method

Statistical analysis was completed on SPSS 23.0 (IBM SPSS statistics, Armonk, NY: IBM Corp) and Graphpad. Differences between groups were compared by Pearson chi-square test (parameter > 5) or Fisher exact test (parameter < 5). Survival analysis of OS and DFS was finished with the Kaplan–Meier method, and differences in survival time were evaluated by log rank test. The risk factors related to OS and DFS were detected using Cox proportional hazards models, and the modeling results were described with 95% CI hazard ratio (HR). *P* < .05 was defined as significant differences. The prognostic evaluation ability of various parameters including preoperative NLR, ALB, LDH, and GRIm score was assessed using receiver’s operating characteristic (ROC) curves. Then the area under the ROC curve (AUC) of each parameter and the difference between AUCs were compared.

## 3. Results

### 3.1. Baseline data

A total of 174 eligible patients from June 2014 to July 2019 were collected. Until the end of follow-up on December 01, 2021, 122 patients died, 2 patients died due to cerebrovascular disease, 52 patients were stable.

All patients obtained the laboratory data of blood routine, biochemical data and clinical and pathological data 1 week before the operation and completed the follow-up evaluation.

Baseline data for all enrolled patients are shown in Table 1.

**Table 1:** Patients’ information.

Characteristics	Model set
Gender	
Male	150
Female	24
Age (yr)	
Range	38–80
Average	63.4
ZPS score	
0-1	158
2	16
Operation	
Transventral	152
Laparoscope	22
Differentiation	
Well	5
Moderately	46
Low	123
Tumor invasion (T-stage)	
T1-2	34
T3-4	140
Lymph node metastasis (N-stage)	
N0-1	74
N2-3	100
pTNM stage	
I–II	72
III	102
Chemotherapy*	
SOX	123
DS	45
Vascular invasion	
Yes	69
No	105
Nerve invasion	
Yes	72
No	102

TNM = tumor, node, metastasis, ZPS = Zubrod – ECOG – performance status.

### 3.2. Effects of different GRIm score on OS and DFS

**Figure 1.**
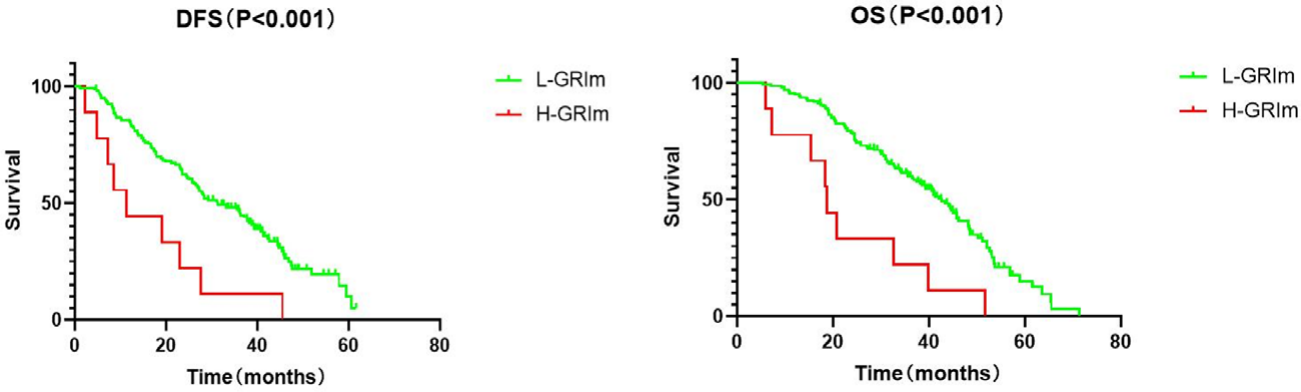
Survival analysis of patients based on GRIm score. DFS = disease-free survival, GRIm Score = Gustave-Roussy Immunity Score, OS = overall survival.

**Figure 2.**
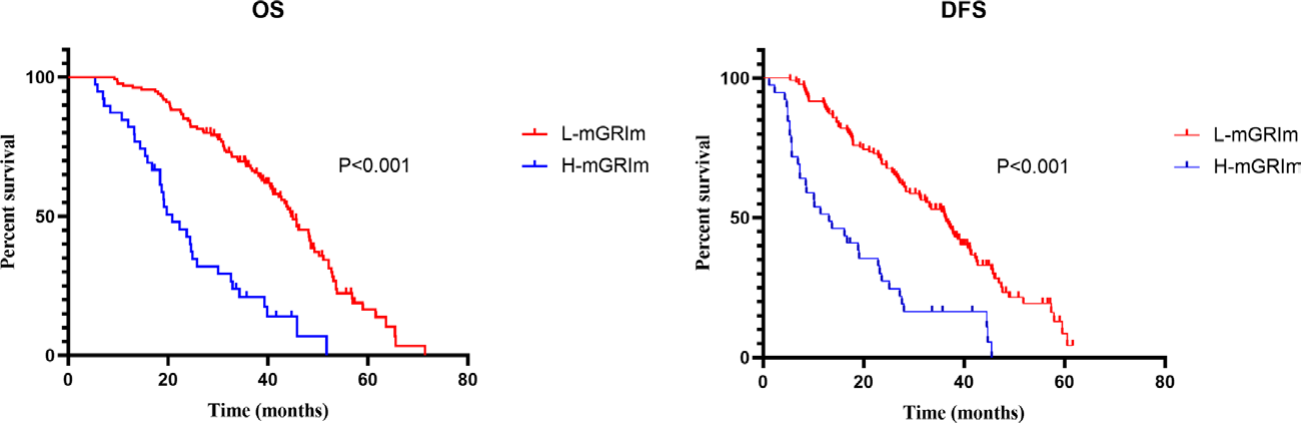
Survival analysis of patients based on mGRIm score. DFS = disease-free survival, mGRIm Score = modified Gustave-Roussy Immunity Score, OS = overall survival.

First, we calculated the GRIm score for patients according to the cutoff value set by Bigot. 166 patients were marked in L-GRIm and 8 patients in H-GRIm groups. By the end of follow-up, all H-GRIm groups had died. The median OS of L-GRIm group was 36.1 and 18.6 months of H-GRIm groups. The median DFS of the L-GRIm and H-GRIm groups was 29.3 and 9.5 months respectively. Both DFS and OS were significantly different between groups (P < .001, P < .001) (Fig. 1). However, according to the Bigot original cutoff value, the number of patients in two groups was obvious, and there was a shift in statistical analysis. Therefore, taking the OS of patients enrolled in this study as the end point, we recalculated the cutoff values of LDH, ALB and NLR by X-tile. And the cutoff value of ALB was 37.3 g/L, of LDH was 193 µ/L and of NLR was 2.48. Then we grouped the patients according to the modified cutoff values. The modified GRIm score was defined as mGRIm. The total mGRIm scores of 0 to 1 and 2 to 3 were recorded as L-mGRIm group (135 patients) and H-mGRIm group (39 patients), respectively. The two groups before operation were sent to Kaplan–Meier survival analysis. The median OS of the H-mGRIm and L-mGRIm groups were 23.2 and 38.6 months, respectively. The median DFS time of the H-mGRIm and L-mGRIm groups was 16.9 and 31.7 months, respectively. Both DFS and OS were significantly different between groups (P = .000, P = .000) (Fig. 2).

### 3.3. The relationship between mGRIm scores and clinicopathological factors

Statistical analysis found that the GRIm score was significantly correlated with the higher TNM stage (*P* < .05), but not with Gender ZPS score, T stage, N stage, Vascular invasion and degree of differentiation (all *P* > .05) (Table 2).

**Table 2:** Pretreatment baseline characteristics, treatment, and laboratory data according to mGRIm Score (N = 174).

Characteristics	L-mGRIm (135)	H-mGRIm (39)	*P*
Gender			
Male	114	36	.55†
Female	21	3	
Age (yr)			
≥65	64	24	.046*
<65	71	15	
ZPS			
0-1	125	33	.19*
2	10	6	
T stage			
T1-2	29	5	.23*
T3-4a	106	34	
N stage			
N0-1	61	13	.06*
N2-3b	74	26	
pTNM stage			
I-II	62	10	**.02** *
IIIA-C	73	29	
Nerve invasion			
Yes	54	18	.12†
No	81	21	
Vascular invasion			
Yes	51	18	.47*
No	84	21	
Differentiation			
Well	5	0	.64†
Moderate/poor	130	39	
NLR			
>2.48	32	36	**.000** †
≤2.48	103	3	
LDH			
>193	26	24	**.000** *
≤193	109	15	
ALB			
≥37.3	125	12	**.000** *
<37.3	10	27	

Bold text indicates a *P* value <.05.

ALB = albumin, LDH = lactate dehydrogenase, mGRIm score = modified-Gustave-Roussy Immunity Score, NLR = neutrophil lymphocyte ratio.

* Chi square test.

† Fisher exact test.

### 3.4. Prognostic value of mGRIm score

#### 3.4.1. Overall survival.

First, we used univariable cox regression to analyze influencing factors of OS for all patients. Results showed that age (*P* = .001), ZPS score (*P* = .000), N stage (*P* = .000), T stage (*P* = .005), TNM stage (*P* = .000), Nerve invasion (*P* = .016), Vascular invasion (*P* = .005), ALB (*P* = .006), NLR (*P* = .000), and GRIm score (*P* = .000) were all significantly correlated with lower OS time. Multivariate Cox regression analysis showed that ZPS (2 vs 0–1: HR 1.99 95% CI 1.05–3.76 *P* = .035), LDH (≥193 vs <193: HR 0.6; 95% CI 0.38–0.95 *P* = .028), mGRIm score (2–3 vs 0–1: HR 2.4; 95% CI 1.09–5.23 *P* = .029) was independent risk factors of OS (Table 3).

**Table 3:** Univariate and multivariate analysis of overall survival based on clinicopathological characteristics.

	Univariable		Multivariable	
Characteristics	HR with 95% CI	*P* value	HR with 95% CI	*P* value
Age (yr)	0.55 (0.38–0.79)	**.001**	0.72 (0.48–1.07)	.1
≤65				
>65				
Gender	1.56 (0.90–2.69)	.11		
Male				
Female				
Differentiation	0.99 (0.67–1.48)	.98		
Well				
Moderately/Low				
ZPS	3.56 (2.07–6.12)	**.000**	1.99 (1.05–3.76)	**.035**
0–1				
2				
Operation	0.73 (0.43–1.23)	.24		
Transventral				
Laparoscope				
Lymph node metastasis (N-stage)	1.96 (1.35–2.84)	**.000**	1.4 (0.85–2.33)	.18
N0-1				
N2-3				
Tumor invasion (T-stage)	1.94 (1.22–3.1)	**.005**	1.49 (0.81–2.75)	.2
T1-2				
T3-4				
TNM stage	2.13 (1.46–3.11)	**.000**	1.05 (0.56–1.95)	.88
I–II				
III				
Nerve invasion	0.6 3 (0.44–0.92)	**.016**	0.97 (0.62–1.53)	.9
Yes				
No				
Vascular invasion	0.59 (0.40–0.95)	**.005**	0.74 (0.48–1.14)	.18
Yes				
No				
ALB	0.56 (0.36–0.85)	**.006**	1.45 (0.79–2.66)	.24
≥37.3				
<37.3				
LDH	0.48 (0.33–0.69)	**.000**	0.6 (0.38–0.95)	**.028**
>193				
≤193				
NLR	0.50 (0.35–0.72)	**.000**	0.92 (0.58–1.46)	.73
>2.48				
≤2.48				
mGRIm score	4.17 (2.7–6.34)	**.000**	2.4 (1.09–5.23)	**.029**
0-1				
2-3				

Bold text indicates a *P* value <.05.

ALB = albumin, LDH = lactate dehydrogenase, mGRIm score = modified -Gustave-Roussy Immunity Score, NLR = neutrophil lymphocyte ratio.

#### 3.4.2. Disease-free survival.

Then, we used univariate cox regression to analyze influencing factors of DFS for all patients. Results showed that age (*P* = .000), ZPS score (*P* = .000), N stage (*P* = .000), T stage (*P* = .006), TNM stage (*P* = .000), Nerve invasion (*P* = .007), Vascular invasion (*P* = .038), ALB (*P* = .008), LDH (*P* = .002), NLR (*P* = .000), and GRIm scores (*P* = .000) were all positive. Multivariate Cox regression analysis showed that the age (>65 vs ≤65 years HR 0.63; 95% CI 0.4–0.95 *P* = .003), LDH (>193 U/L vs ≤193 U/L: HR 0.55; 95% CI 0.37–0.82 *P* = .004), and mGRIm score (2–3 vs 0–1: HR 4.74; 95% CI 2.24–9.9 *P* = .000) as an independent risk factor for DFS (Table 4).

**Table 4:** Univariate and multivariate analysis of disease-free survival based on clinicopathological characteristics.

	Univariate		Multivariate	
Characteristics	HR with 95% CI	*P* value	HR with 95% CI	*P* value
Age (yr)	0.51 (0.35–0.74)	**.000**	0.63 (0.4–0.95)	**.03**
≤65				
>65				
Gender	1.42 (0.82–2.45)	.21		
Male				
Female				
Differentiation	0.93 (0.62–1.38)	.93		
Well				
Moderately/Low				
ZPS	2.76 (1.62–4.72)	**.000**	1.24 (0.66–2.33)	.49
0-1				
2				
Operation	0.83 (0.49–1.42)	.50		
Transventral				
Laparoscope				
Lymph node metastasis (N-stage)	1.96 (1.35–2.84)	**.000**	1.5 (0.93–2.5)	.09
N0-1				
N2-3				
Tumor invasion (T-stage)	1.97 (1.2–3.2)	**.006**	1.47 (0.78–2.74)	.23
T1-2				
T3-4				
TNM stage	2.07 (1.4–3.02)	**.000**	0.95 (0.51–1.76)	.87
I–II				
III				
Nerve invasion	0.6 (0.4–0.87)	**.007**	0.73 (0.45–1.17)	.19
Yes				
No				
Vascular invasion	0.68 (0.47–0.98)	**.038**	0.95 (0.59–1.5)	.82
Yes				
No				
ALB	0.56 (0.37–0.86)	**.008**	1.76 (0.89–3.48)	.1
≥37.3				
<37.3				
LDH	0.55 (0.38–0.79)	**.002**	0.55 (0.37–0.82)	**.004**
>193				
≤193				
NLR	0.53 (0.37–0.75)	**.000**	1.15 (0.71–1.87)	.56
>2.48				
≤2.48				
mGRIm score	3.55 (2.36–5.35)	**.000**	4.74 (2.24–9.96)	**.000**
0-1				
2-3				

Bold text indicates a *P* value <.05.

ALB = albumin, LDH = lactate dehydrogenase, mGRIm score = modified -Gustave-Roussy Immunity Score, NLR = neutrophil lymphocyte ratio.

### 3.5. Efficacy test of prognostic evaluation of GRIm score

**Figure 3.**
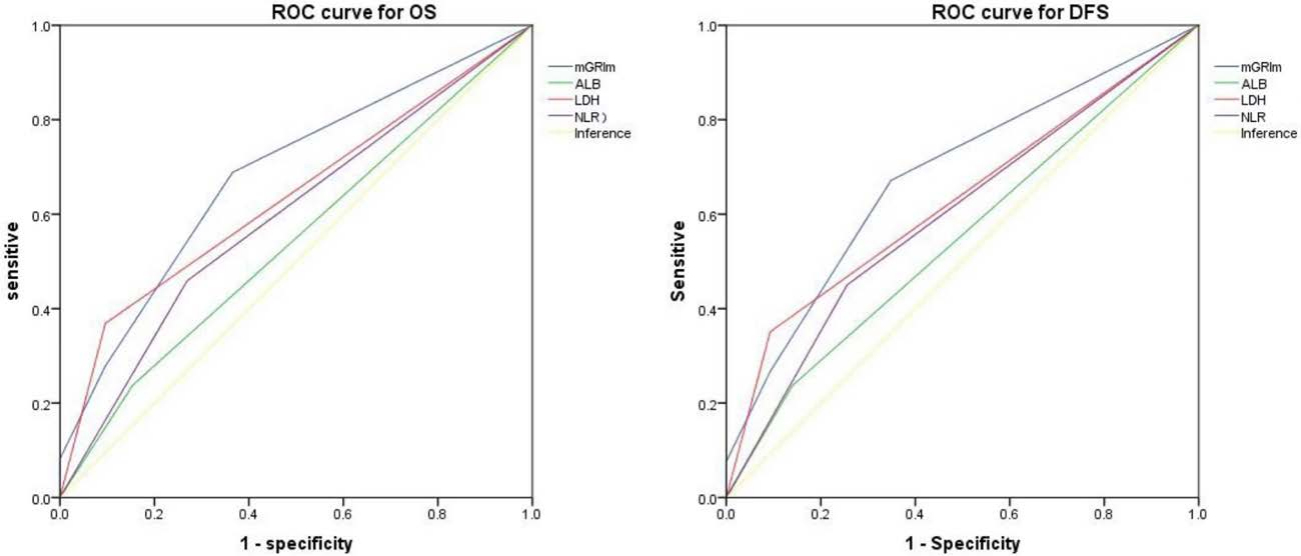
Differences in the ROC curves for the three parameters and the mGRIm scores. DFS = disease-free survival, mGRIm Score = modified Gustave-Roussy Immunity Score, ROC = receiver’s operating characteristic, OS = overall survival.

The ROCs of ALB, LDH, NLR, and mGRIm score in the diagnostic efficacy of resectable proximal GC were further studied. Follow-up cutoff, death and relapse was taken as the dependent variable to conduct ROC curve analysis for OS and DFS. According to the ROC analysis for the patients, the AUCs of OS and DFS predicted by mGRIm score were 0.68 (95% CI 0.6–0.7 *P* = .000) and 0.68 (95% CI 0.59–0.77 *P* = .000), of ALB were 0.54 (95% CI 0.45–0.63 *P* = .38) and 0.55 (95% CI 0.45–0.65 *P* = .34), of LDH were 0.64 (95% CI 0.55–0.72 *P* = .004) and 0.63 (95% CI 0.54–0.72 *P* = .011), of NLR were 0.6 (95% CI 0.51–0.69 *P* = .05) and 0.6 (95% CI 0.5–0.69; *P* = .06) respectively. In conclusion, it can be seen that the AUC area of GRIm is larger than the other three parameters, which means its prognostic evaluation efficiency is better than that of the three parameters alone (Fig. 3).

## 4. Discussion

NLR reflects the inflammatory state, which is the clinical stage and long-term survival of patients with malignant tumors, including GC.^[20]^ Preoperative NLR is a highly reproducible, cost-effective and widely-used marker for long-term postoperative prognosis of GC patients.^[21–23]^ Miyamotor et al^[22]^ analyzed the prognostic values of preoperative NLR in 154 stage II/III GC patients receiving surgical treatments, and confirmed that preoperative NLR > 3.5 was connected with both long-term and perioperative prognosis of GC patients. The research conducted by Yamada et al^[24]^ showed that NLR > 2.5 was a detached prognostic factor of OS in the prognostic evaluation of 89 cases of advanced GC. Elevated serum LDH level had been proven to be associated with poor prognosis in such some tumors as melanoma, gastrointestinal malignancies,GC, head and neck tumors and lung cancer.^[25–31]^ Wang et al^[29]^ concluded that LDH above the cutoff value of 157.2 µ/L was significantly correlated with poor OS (HR = 1.73, 95% CI 1.16–2.57) and DFS (HR = 1.74, 95% CI 1.30–2.40) in 619 resectable GC patients. As for the relationships between preoperative LDH level and prognosis, Zhao et al found that preoperative LDH greater than the normal reference ranges suggested worse OS and PFS in 365 GC patients.^[28]^ The ALB level, as an important nutritional factor, reflects the nutritional status of patients.^[7]^ Moreover, preoperative serum ALB level is reportedly a clinical prognostic indicator of cancers.^[32,33]^ According to a relevant report, ALB is a separate prognostic factor of operable GC through the systemic inflammation score constructed when ALB is selected at 40 g/L.^[7]^ When the cutoff value of ALB is 34.9 g/L, a lower ALB level reflects a poor prognosis (HR: 1.40, 95% CI: 1.14–1.13).^[34]^

Bigot et al constructed the GRIm score based on LDH, NLR, and ALB was first proposed to better select patients in clinical trials of immunotherapy, and results showed that the GRIm score was a better prognostic index for patients included in the experimental trials.^[15,18]^ In recent years, the prognostic values of GRIm scores in non-small cell lung cancer,^[35]^ small cell lung cancer^[36]^ and esophageal squamous cell carcinoma^[18]^ were improved. It was confirmed that high GRIm score was associated with poor OS and DFS (PFS). However, regarding the effects of GRIm scores on proximal GC, there are few studies on the prognostic value. Therefore, the purposes of this study were to retrospectively analyze the impacts of preoperative GRIm scores on the prognosis of proximal GC patients and to clarify the potential values of the GRIm scores. The GRIm score was used to analyze its role in 174 cases of resectable proximal GC treated with radical total gastrectomy. The patients were divided into L-GRIm group (166 patients) and H-GRIm group (8 patients). Kaplan–Meier survival analysis suggested that the median OS of L-GRIm group was 36.1 and 18.6 months of H-GRIm groups. The median DFS time of the L-GRIm and H-GRIm groups was 29.3 and 9.5 months respectively. Both DFS and OS were significantly different between groups (*P* < .001, *P* < .001). We also confirmed high-GRIm scores (2–3 vs 0–1: HR 3.16; 95% CI 1.23–10.3 *P* = .019) were independent risk factors of OS and high-GRIm score (2–3 vs 0–1: HR 3.88; 95% CI 1.71–8.79 *P* = .001) as a free-standing risk factor for DFS. Although the original cutoff value grouping has been verified that the GRIm score can be used as the prognosis evaluation index of OS and DFS, the number of patients in the two groups is different, which may have statistical bias, so in order to make the results of this study more credible, the researchers adopted it ROC curve, X-tile analysis, cutoff inder application, and the median or normal value range specified by the research unit are mostly used to determine the cutoff value. Based on previous research,^[37–39]^ we used X-tile to recalculate the cutoff values of the three parameters. According to the survival data, the cutoff values of ALB, LDH, and NLR determined on X-tile were 37.3 g/L, 193 µ/L and 2.48 respectively, which were used to build mGRIm. Modified GRIm divided the patients into the H-mGRIm and L-mGRIm groups, Analysis showed that H-mGRIm was associated with poor OS (23.2 vs 38.6) months and DFS (16.9 and 31.7) months which are consistent with previous studies.^[18,33,37,40]^ The reason why mGRIm outperformed Bigot’s GRIm may be that we used X-tile to determine the best cutoff values of three parameters for the prognosis evaluation of gastric cancer, and accurately grouped mGRIm according to the survival curve of each group. Through univariate and multivariate regression analysis, mGRIm score was an independent factor affecting OS (2–3 vs 0–1: HR 2.4; 95% CI 1.09–5.23 *P* = .029) and DFS (2–3 vs 0–1: HR 4.74; 95% CI 2.24–9.9 *P* = .000). Consistent with it is the Seigo Minami.^[16]^ Studies in small cell lung cancer have found that OS was significantly shorter in high GRIm-score group than in low group (median 6.1 vs 11.4 months, *P* < .01). They also confirmed the high GRIm-score (HR 1.80, 95% CI 1.20–2.72, *P* < .01), as separate and prognostic factors of OS. Li et al^[17]^ In non-small cell lung cancer undergoing thoracoscope surgery in the study of remained negligible that both the OS and DFS were significantly shortened along with each number increase in the GRIm-score group, showing a step-wise fashion (log-rank *P* < .001).

In addition, we study the role of such three parameters as ALB, NLR, LDH, and GRIm scores in prognosis assessment, and found that the mGRIm score predicted the OS and DFS through ROC curve the AUCs area were 0.62 (95% CI 0.58–0.85 *P* = .031) and 0.62 (95% CI 0.56–0.85 *P* = .016), larger than the other three parameters. But its AUC curve area is less than 0.75, the predictive value is undesirable. However, this is consistent with the findings of previous studies. The Feng et al^[18]^ study of the GRIm score in the prognostic assessment role of resectable esophageal cancer found that the AUC area of GRIm was 0.64, which is consistent with the results of this study. Besides, Schnöller et al^[40]^ investigated the prediction effects of the GRIm scores in limited-stage small cell lung cancer, found that the GRIm scores did not have a prognostic prediction effect. Since GRIm has been insufficiently studied in proximal GC, the results of this study still deserves further confirmation.

To the best of our knowledge, this study is the first to show the prognostic value of GRIm score in resectable proximal GC patients. This study reveals several important findings. First of all, we demonstrates the GRIm score can be used to evaluate the OS and DFS of resectable proximal GC patients. Secondly, the GRIm score, rather than NLR, ALB, or LDH, is a useful independent prognostic factor. In conclusion, the mGRIm score is not only a selective biomarker for patients included in immunotherapy clinical trials, but also a useful prognostic biomarker for proximal GC patients undergoing surgical resection. However, this study also has some limitations. First, the data are from a single-center retrospective study, which may have bias. Second, the number of patients in the study was small and few reports in previous GC patients, so our results still need to be further confirmed by large-sample prospective studies. Third, the unconventional cut value mGRIm score may limit the interpretability and extrapolation of the results, and prospective studies with large samples are expected to further explore the application value and clinical significance of mGRIm Score in the prognosis of GC patients. Considering the possible impact of postoperative complications on prognosis, we will conduct further research. In conclusion, the preoperative mGRIm score greater than 1 is a risk factor affecting the long-term postoperative survival. Clinically, it can be used as one of the factors to judge prognosis and guide treatment.

## 5. Conclusions

Age is an independent prognostic factor for resectable proximal GC and should be strictly considered in treatment allocation. Also, the values of mGRIm score before operation proximal GC were studied. mGRIm score is a novel, simple and effective index for prognosis evaluation of resectable proximal GC. We expect large-sample prospective studies to further evaluate the application values and clinical significance of mGRIm score in proximal GC patients.

## Author contributions

**Conceptualization:** Yujing Shi, Xinchen Sun, Liang Liang, Xiaoke Di.

**Data curation:** Yujing Shi, Mengyang Ju, Chenhong He, Liang Liang.

**Formal analysis:** Yujing Shi, Mengyang Ju, Chenhong He, Liang Liang.

**Funding acquisition:** Yujing Shi, Liang Liang.

**Investigation:** Liang Liang.

**Methodology:** Yujing Shi, Xiaojiao Chen, Liang Liang, Xiaoke Di.

**Software:** Yujing Shi.

**Supervision:** Xinchen Sun.

**Validation:** Mengyang Ju, Xiaojiao Chen, Xiaoke Di.

**Visualization:** Xinchen Sun, Xiaojiao Chen, Liang Liang.

**Writing – original draft:** Yujing Shi.

**Writing – review & editing:** Xinchen Sun, Xiaojiao Chen, Chenhong He, Liang Liang, Xiaoke Di.
